# Childbirth experiences of women with a history of physical, sexual, and child abuse: a cross-sectional study of 2,575 Russian women

**DOI:** 10.1186/s12884-024-06369-3

**Published:** 2024-03-08

**Authors:** Anna Suarez, Vera Yakupova

**Affiliations:** Department of Psychology, Lomonosov Moscow State University, Mokhovaya St, 11/9, Moscow, 125009 Russia

**Keywords:** Childbirth, Exposure to violence, Physical abuse, Sexual trauma, Child abuse, Perinatal care, Obstetric labor complications

## Abstract

**Background:**

A substantial number of women who subsequently become pregnant and give birth have a history of physical, sexual, and/or child abuse. This study investigated the associations of these types of traumas and their cumulative effect with childbirth experiences, namely, mode of birth, maternal and child complications during pregnancy/childbirth, preterm birth, medical procedures, and obstetric violence during labour.

**Methods:**

A group of Russian women (*n* = 2,575) who gave birth within the previous 12 months, completed a web-based survey, where they provided demographic information, details about their childbirth experiences, and a history of trauma.

**Results:**

Women with any type of past abuse were at higher risk for maternal complications during pregnancy/childbirth (exp(β) < 0.73, *p* < 0.010 for all). More specific to the type of trauma were associations of physical abuse with caesarean birth, child abuse with complications during pregnancy/childbirth for the baby, and physical and child abuse with obstetric violence (exp(β) < 0.54, *p* < 0.022 for all). There was a cumulative effect of trauma for all the outcomes except for medical procedures during childbirth and preterm birth.

**Conclusion:**

This study provides insights into potential different individual effects of physical, sexual, and/or child abuse as well as their cumulative impact on the childbirth experiences. The robust findings about maternal complications during pregnancy/childbirth and obstetric violence highlight the importance of trauma-informed care, supportive policies, and interventions to create safe and empowering birthing environments that prioritise patient autonomy, dignity, and respectful communication.

## Background

Nearly 1 in 3 women across the globe have experienced physical and/or sexual violence by an intimate partner or non-partner violence, or both [1]. A recent worldwide meta-analysis further discovered an overall pooled child sexual abuse prevalence of 24% [2], which is slightly higher than World Health Organization (WHO) previous estimates of one in five women having experienced sexual abuse as a child [3]. Taken together, these dark statistics show that a substantial number of women who subsequently become pregnant and give birth have a history of physical, sexual, and/or child abuse.

While some women with a history of abuse find childbirth to be a positive and healing experience, research suggests that for many others maternity care can trigger feelings of violation and mistreatment similar to their experiences of abuse in the past [4]. In a qualitative study carried out by Halvorsen and colleagues, women who had experienced rape in adulthood revealed that the act of giving birth was comparable to “being back in the rape” for them [5]. Indeed, many characteristics of childbirth and labour, such as pain, invasive pelvic examinations, restriction of movement, body position, and the inequality of relationship between a woman and the healthcare providers may trigger flashbacks, sense of objectification, and loss of control [6, 7]. Furthermore, childbirth experiences have been associated with acute stress and dissociative responses in survivors of sexual assault and child sexual abuse [5].

There is a significant body of literature indicating that previous traumatic events may predispose to posttraumatic stress disorder (PTSD), which is also true for childbirth-related PTSD (CB-PTSD) [8, 9]. Seng and colleagues showed that pregnant women with PTSD were five times more likely to have experienced completed child rape than those who did not have PTSD [10]. Similarly, Oliveira et al. indicate that among women who developed CB-PTSD, approximately 30.2% reported a history of child sexual abuse, 92.5% reported experiencing psychological abuse and 45% - physical violence from their partners during pregnancy [11].

Despite this convincing evidence, the pathways underlying these associations remain unclear. Multiple studies found that a woman’s perception of the severity of the event is more significant than the objective stressor’s actual severity [12, 13]. An uncomplicated birth may be perceived by a woman as a negative or even traumatic experience; conversely, labour with serious complications may be generally perceived positively [14]. As Cheryl Tatano Beck defines it ‘trauma lies in the eye of the beholder’ [15]. These findings are in line with the results of several recent overviews showing that one of the most critical predictors of CB-PTSD is a woman’s negative subjective experience of childbirth [16, 17].

On the other hand, a significant body of literature indicates that women with a history of sexual assault or child maltreatment have higher rates of miscarriage, obstetric complications, preterm delivery, and unplanned caesarean birth (CB) as compared to women without such history [6, 18]. Furthermore, a retrospective cohort study of over 2 million Canadian women showed that women who were hospitalized for physical or sexual assault, and assault with documented intimate partner violence before and during pregnancy were at higher future risk of placental abruption, antepartum haemorrhage, stillbirth, preterm birth, and low birthweight [19]. Contrarily, other studies found no such differences [20, 21].

Montgomery and colleagues further highlight that it might be the way the medical procedures are performed that trigger memories of abuse rather than them per se [4]. A ‘re-enactment of abuse’ may come from actions and words that diminish a birthing woman’s sense of control and disempower her, which may come in a form of obstetric violence [4, 22], which, in turn, is often reported as another major risk factor for CB-PTSD [23, 24]. Bohren et al. show that women often felt stripped of their dignity as a result of objectification of their bodies by healthcare providers; numerous, painful pelvic examinations during labour were perceived as dehumanising [25]. The survivors of physical, sexual, and/or child abuse may further experience higher rates of disrespect and abuse during labour, as it has been well documented that early abuse is a risk factor for adult victimisation in women [26, 27]. However, to date, there is a lack of evidence of the association between a history of physical, sexual, and/or child abuse and experience of obstetric violence and its types. Moreover, although there is also an indication from a recent systematic review that exposure to different types of maltreatment during childhood influenced the risk of adverse perinatal outcomes in differing ways [18], the studies of different types of lifetime adversity’s effects on childbirth experiences remain scarce.

In our recent study of the present cohort we found that women with past experience of physical, sexual, and/or child abuse had higher symptoms of CB-PTSD [9]. Furthermore, the more traumatic experiences women had in the past, the higher were CB-PTSD scores. However, it is unclear whether it is related to the differences in the childbirth experiences of women with and without a history of physical, sexual, and/or child abuse which present risk for developing CB-PTSD, or rather differences in the experiences of relationships with the healthcare providers.

While it is not always possible to predict and prevent medical complications during pregnancy and/or childbirth, the use of some medical procedures during birth is avoidable or there are various ways for them to be applied and communicated to the women, particularly in the presence of maternal history of abuse. As women with previous traumatic experiences are shown to be at higher risk for developing CB-PTSD [4, 6, 9–11], it is important to investigate what characteristics of childbirth may elevate this risk, specifically if they can be modified via respectful trauma-informed maternity care. Nevertheless, to date, there have been no studies exploring the associations between a history of abuse and common medical procedures during birth as well as experiences of obstetric violence from the care providers. Moreover, it is understudied whether women with different types of abuse experiences in the past are more sensitive to different medical procedures and ambiguous behaviours from the midwives and other caregivers and whether these experiences work cumulatively.

Therefore, to address the gaps in the literature and explore potential avenues for prevention of CB-PTSD among women with traumatic past experiences, this work aims to investigate the association between a history of physical, sexual, and/or child abuse and childbirth experiences. Namely, we will focus on the mode of birth, complications for the woman or her child during pregnancy and/or childbirth, preterm birth, medical procedures during childbirth and their types, and experience of obstetric violence and their types as the outcomes. Moreover, we will focus both on the individual types of past traumatic experiences and cumulative trauma. Based on the previous studies, we hypothesise that there are higher risks of all adverse childbirth outcomes among women with a history of abuse.

## Methods

### Participants

This cross-sectional study comprises the data collected between May and September in 2022. Women were invited to participate in the web-based survey via social media (relevant Instagram, Facebook and VK communities and perinatal health professional pages), antenatal classes, and classes for new parents. Invitations were also delivered by the doctors and midwives in maternity hospitals and healthcare clinics. We received 2,954 responses. The participants were included in the study if they were older than 18, could read and type in Russian, and gave birth to a live-born child within the previous 12 months in Russia. In total, 2,575 fulfilled the inclusion criteria and formed the final sample of this study.

### Measures

#### Demographic characteristics

The survey included questions about the participants’ age at the time of childbirth, highest achieved level of education (primary/secondary/tertiary), marital status (married/in relationship/single), and perceived socioeconomic status in comparisons to other residents of their current region (low/middle/high).

#### Childbirth experience and obstetric characteristics

Respondents answered questions regarding their pregnancy and childbirth experiences, namely parity, gestational age at childbirth, number of months from the childbirth to the date of filling in the survey, and mode of birth (vaginal/ assisted vaginal (with the use of forceps/vacuum)/ emergency caesarean/ planned caesarean).

Furthermore, they indicated whether and what kind of medical procedures had been performed during labour (none/amniotomy/use of synthetic oxytocin/epidural analgesia/episiotomy/other) and whether and what kind of obstetric violence experiences had occurred during labour (none/bullying and verbal abuse/threats and accusations/medical procedures without consent/denial of pain relief/use of Kristeller manoeuvre/other). A sum of the total number of medical procedures as well as total number of instances of obstetric violence during labour were calculated, ranging from 0 (none) to 5 (all) and 0 (none) to 6 (all), respectively.

Finally, women reported whether they experienced medical complications during pregnancy or childbirth either in relation to their own health (none/minor/major) or the health of their baby (none/minor/major).

#### Past traumatic experience

The survey further included a list of potentially traumatic life events (Serious, life threatening illness / Physical abuse / Sexual abuse / Military combat or lived in a war zone / Child abuse / Accident / Natural disaster / Other trauma). The participants were asked to tick the boxes next to the events they had experienced or witnessed at least once at some point of their lives. For the purposes of this study, we focused on the traumatic experiences that were previously associated with CB-PTSD in this cohort [9], namely Physical abuse (e.g., attacked with a weapon, severe injuries from a fight, held at gunpoint, etc.), Sexual abuse (e.g., attempted rape, forced sexual act with a weapon, etc.), and Child abuse (e.g., severe beatings, sexual acts with someone 5 years older than you, etc. before age 18). We further calculated the sum of lifetime traumatic types women experienced and categorised them into four groups, including 0 = never experienced, 1 = exposure to one type, 2 = exposure to two types, 3 = exposure to all three types (i.e., physical abuse, sexual abuse, and child abuse). The exposure to each lifetime traumatic type (i.e., physical abuse, sexual abuse, and child abuse) is defined as 0 = never exposed to any, and 1 = exposed to at least one of action related to the traumatic type.

#### Covariates

All analyses were adjusted for maternal and obstetric characteristics previously associated with past traumatic or childbirth experiences as covariates: maternal age at the time of childbirth, level of education, marital status, and perceived socioeconomic status as well as gestational age at birth, parity, and number of months from the childbirth to the date of filling in the survey. In the analysis exploring the association with preterm birth, gestational age was excluded from the list of covariates.

### Statistical analyses

The proportional odds model was used to explore the associations between the individual types of past traumatic experiences and mode of birth, childbirth complications, preterm birth, types of medical procedures, and types of obstetric violence experiences.

Binary logistic regression tested the association of the number of lifetime traumatic types experienced with the mode of birth (vaginal/caesarean) and preterm birth (yes/no).

Ordinal logistic regression analysis was performed to assess the association between the number of lifetime traumatic types experienced and medical complications during pregnancy/childbirth (none/minor/major).

We explored the association of the individual types of past traumatic experiences with the number of medical procedures and obstetric violence experiences using generalised linear models.

Finally, multiple linear regression tested the associations between the number of lifetime traumatic types experienced and the number of medical procedures and instances of obstetric violence experienced during labour.

The level of significance was set to *α* = 0.05. All analyses were performed using SPSS 28 software.

## Results

Demographic, childbirth, and trauma-related characteristics for participants are presented in Table 1. It shows that most participants were highly educated (91.1%), married (91.7%), and had average income in comparison to other families from their region (66.2%). For most participants it was the first child (63.1%), on average born vaginally (73%) at term (Mean = 39.57, Standard Deviation = 1.65). A substantial number of participants reported having experienced complications during pregnancy or childbirth in relation to their own health (44.6%), while complications in relation to baby’s health occurred for 19.1% of women. Absolute majority of the participants had at least one medical procedure during labour (82%), their quantity ranging from one (*n* = 1022, 48.2%) to five (*n* = 6; 0.3%). Almost a third of women reported at least one instance of obstetric violence during labour (31.5%), the number of instances among them ranging from one (*n* = 531, 63.8%) to six (*n* = 1, 0.1%).

**Table 1:** Characteristics of the sample (*n* = 2,575)

Characteristic		Mean/Median/N	SD/%	Range
Maternal age		31.03	4.29	18-46
Education	Primary	44	1.7%	
	Secondary	184	7.1%	
	Tertiary	2347	91.2%	
Family status	Married	2362	91.7%	
	In relationship	151	5.9%	
	Single	51	2%	
Perceived socioeconomic status	Low-income	263	10.2%	
	Middle-income	1704	66.2%	
	High-income	608	23.6%	
Region of childbirth facility	Moscow	588	22.8%	
	St.Petersburg	289	11.2%	
	Another region in Russia	1698	66%	
Parity	1	1626	63.1%	
	2	699	27.1%	
	3+	250	9.7%	
Number of months from the childbirth to the date of filling in the survey		5.49	3.52	0-12
Gestational age at birth(weeks)		39.57	1.65	25-43
Preterm birth (yes)		99	3.8%	
Mode of birth	Vaginal	1879	73%	
	Assisted vaginal (with the use of forceps/vacuum)	64	2.5%	
	Emergency caesarean	406	15.7%	
	Planned caesarean	226	8.8%	
Medical complications during this pregnancy/childbirth (mother)	None	1428	55.4%	
	Minor complications	1006	39.1%	
	Major complications	141	5.5%	
Medical complications during this pregnancy/childbirth (baby)	None	2082	80.9%	
	Minor complications	415	16.1%	
	Major complications	78	3.0%	
At least one medical procedure during labour (yes)		2111	82%	
Type of medical procedures during labour	Amniotomy	1081	41.9%	
	Use of synthetic oxytocin	721	28%	
	Epidural analgesia	975	37.8%	
	Episiotomy	481	18.7%	
	Other medical procedure	501	19.4%	
Total number of medical procedures		1.00	1.05	0-5
At least one instance of obstetric violence during labour (yes)		809	31.4%	
Type of instance of obstetric violence experienced during labour	Bullying and verbal abuse	377	14.6%	
	Threats and accusations	172	6.7%	
	Denial of pain relief	129	5%	
	Medical procedures without consent	206	8%	
	Kristeller manoeuvre	165	6.4%	
	Other types of obstetric violence	233	9%	
Total number of instances of obstetric violence experienced during labour		0.00	0.87	0-6
At least one type of lifetime trauma experienced (yes)		690	26.8%	
Type of lifetime traumatic experience	Physical abuse	205	7.9%	
	Sexual abuse	281	10.9%	
	Child abuse	425	16.5%	
Total number of lifetime traumatic types experienced		1.00	1.05	0-3

### Past trauma and mode of birth

We found that women who experienced physical abuse gave birth via caesarean significantly more often than those without such experience (exp(β) = 0.66, *p* = 0.011). Table 2 shows that this is also true when we look at the mode of birth in four categories (vaginal/ assisted vaginal (with the use of forceps/vacuum)/ emergency caesarean/ planned caesarean). There were no such associations for women with a history of sexual or child abuse (Table 2).

**Table 2:** Association of a history of physical, sexual and/or child abuse and childbirth experiences

	Statistical parameters	Physical abuse	Sexual abuse	Child abuse
Mode of birth (vaginal/caesarean)	Exp(β)	**0.66**	0.84	0.92
	p-value	**0.011**	0.25	0.54
	95% CI	**0.48–0.91**	0.63–1.13	0.72–1.19
Mode of birth (vaginal/ planned caesarean/ assisted vaginal (with the use of forceps/vacuum)/ emergency caesarean)	Exp(β)	**0.7**	0.92	1.0
	p-value	**0.021**	0.54	0.97
	95% CI	**0.51–0.95**	0.69–1.21	0.78–1.27
Medical complications during this pregnancy/childbirth (mother) (none/minor/major)	Exp(β)	**0.61**	**0.73**	**0.73**
	p-value	**p < 0.001**	**0.01**	**0.003**
	95% CI	**0.46–0.81**	**0.57–0.93**	**0.59–0.9**
Medical complications during this pregnancy/childbirth (baby) (none/minor/major)	Exp(β)	0.78	0.8	**0.76**
	p-value	0.17	0.15	**0.037**
	95% CI	0.55–1.11	0.58–1.09	**0.59–0.98**
Preterm birth (yes/no)	Exp(β)	0.81	1.44	0.77
	p-value	0.55	0.33	0.99
	95% CI	0.40–1.63	0.69–3.02	0.54–1.58
At least one medical procedure during labour (yes/no)	Exp(β)	0.81	0.92	0.83
	p-value	0.31	0.62	0.22
	95% CI	0.54–1.22	0.65–1.29	0.62–1.11
Amniotomy (yes/no)	Exp(β)	1.22	1.07	0.91
	p-value	0.20	0.63	0.40
	95% CI	0.9–1.65	0.82–1.39	0.73–1.13
Use of synthetic oxytocin (yes/no)	Exp(β)	1.29	0.88	0.87
	p-value	0.14	0.36	0.26
	95% CI	0.92–1.82	0.66–1.16	0.69–1.11
Epidural analgesia (yes/no)	Exp(β)	0.89	0.94	0.99
	p-value	0.46	0.67	0.94
	95% CI	0.66–1.21	0.72–1.23	0.79–1.24
Episiotomy (yes/no)	Exp(β)	1.41	1.43	1.11
	p-value	0.10	0.053	0.48
	95% CI	0.94–2.11	0.99–2.07	0.83–1.46
At least one instance of obstetric violence during labour (yes/no)	Exp(β)	**0.69**	0.8	**0.55**
	p-value	**0.014**	0.10	**p < 0.001**
	95% CI	**0.51–0.93**	0.61–1.05	**0.44–0.69**
Bullying and verbal abuse (yes/no)	Exp(β)	**0.54**	0.78	**0.55**
	p-value	**p < 0.001**	0.15	**p < 0.001**
	95% CI	**0.38–0.76**	0.56–1.09	**0.42–0.72**
Threats and accusations (yes/no)	Exp(β)	0.66	0.8	**0.65**
	p-value	0.10	0.37	**0.022**
	95% CI	0.4–1.08	0.5–1.29	**0.44–0.94**
Denial of pain relief (yes/no)	Exp(β)	1.05	0.88	**0.59**
	p-value	0.89	0.66	**0.013**
	95% CI	0.54–2.05	0.5–1.55	**0.38–0.89**
Medical procedures without consent (yes/no)	Exp(β)	0.72	0.67	0.72
	p-value	0.17	0.057	0.072
	95% CI	0.45–1.15	0.44–1.01	0.51–1.03
Kristeller manoeuvre (yes/no)	Exp(β)	1.01	0.99	0.84
	p-value	0.97	0.95	0.40
	95% CI	0.57–1.8	0.59–1.65	0.56–1.26

*Note* Exp(β) stands for the β coefficients that express the relative risk or log odds of an outcome being equal to one value versus the other value ln(P(outcome="no”)/P(outcome="yes”)) = intercept + β1*type of abuse + β2*maternal age at the time of childbirth + β3*maternal level of education + β4 *marital status + β5*perceived socioeconomic status + β6*gestational age at birth + β7*parity + β8* number of months from the childbirth to the date of filling in the survey. In the analyses with complications during childbirth the exp(β) coefficients express the values from the formula with two intercepts: ln(P(outcome="none”)/P(outcome="minor”)) = intercept1 + β1*type of abuse + β2*maternal age at the time of childbirth + β3*maternal level of education + β4 *marital status + β5*perceived socioeconomic status + β6*gestational age at birth + β7*parity + β8* number of months from the childbirth to the date of filling in the survey; and ln((P(outcome="none”) + P(outcome="minor”))/P(outcome="major”)) = intercept2 + β1*type of abuse + β2*maternal age at the time of childbirth + β3*maternal level of education + β4 *marital status + β5*perceived socioeconomic status + β6*gestational age at birth + β7*parity + β8* number of months from the childbirth to the date of filling in the survey. In the analysis exploring the association with preterm birth, gestational age was excluded from the list of covariates. 95% CI stands for 95% confidence interval

Binary regression analysis showed that after adjustment for covariates there was a significant association between the number of lifetime traumatic types experienced and mode of birth (Table 3).

**Table 3:** Associations of the number of lifetime traumatic types experienced and childbirth outcomes

Outcome	OR/B	95% CI	p-value
Mode of birth	1.16	1.01; 1.33	0.039
Medical complications during this pregnancy/childbirth (mother)	1.28	1.14;1.44	< 0.001
Medical complications during this pregnancy/childbirth (baby)	1.19	1.03;1.38	0.016
Preterm birth	0.98	0.71;1.34	0.89
Number of medical procedures during labour	0.017	-0.043;0.077	0.58
Number of instances of obstetric violence experienced during labour	0.12	0.07;0.17	< 0.001

*Note* OR stands for the Odd Ratio coefficient from the binary logistic or ordinal logistic regression models exploring the mode of birth, medical complications during pregnancy/childbirth, and preterm birth as outcomes. B stands for unstandardized regression coefficient from the multiple linear regression models exploring the number of medical procedures and instances of obstetric violence experienced during labour as outcomes. 95% CI stands for 95% Confidence Interval. All models are adjusted for maternal age at the time of childbirth, level of education, marital status, and perceived socioeconomic status as well as gestational age at birth, parity, and number of months from the childbirth to the date of filling in the survey. In the analysis exploring the association with preterm birth, gestational age was excluded from the list of covariates

### Past trauma and childbirth complications

Table 2 further shows that experiences of any one of the three types of abuse were associated with higher risk of complications during pregnancy and/or childbirth for the mother, while only child abuse experience remained significant in relation to complications for the baby’s health (exp(β) = 0.76, *p* = 0.037).

Furthermore, ordinal logistic regression analyses of the number of lifetime traumatic types experienced in relation to complications during pregnancy/childbirth demonstrated that experience of each additional type of abuse increased the probability of more serious complications for the mother by 28% (Table 3). Similarly, babies of women with more traumatic experiences were at higher risk of complications during pregnancy or childbirth (Table 3).

### Past trauma and preterm birth

There were no statistically significant associations between having experienced any type of trauma in the past and having a preterm birth (p-values for all > 0.33) (Table 2). Binary logistic regression also did not find association between the number of lifetime traumatic types experienced and higher risk of preterm birth (Table 3).

### Past trauma and medical procedures

Table 2 shows that there were no significant associations between any of the types of the past traumatic experiences and any of the types of medical procedures (p-values for all > 0.053). Furthermore, the number of lifetime traumatic types experienced was not associated with the number of medical procedures during labour (Table 3).

### Past trauma and obstetric violence during childbirth

Table 2 further demonstrates that women who experienced or witnessed physical or child abuse had a significally higher risk for encountering at least one type of obstetric violence. Namely, women from both of these groups were at higher risk for experiencing bullying and verbal abuse, while only women with a history of child abuse had a higher risk for threats and accusations and denial of pain relief (Table 2). None of these associations were discovered for a history of sexual abuse. There were no significant associations in relation to medical procedures without consent and use of Kristeller manoeuvre as outcomes for any type of past trauma (p-values for all > 0.057).

Finally, multiple linear regression analysis revealed that the more types of lifetime traumatic experiences the woman had had in the past, the more instances of obstetric violence occurred during her labour (Table 3).

## Discussion

This work sheds light on the characteristics of pregnancy and childbirth experiences of women with a history of physical, sexual, and/or child abuse. We discovered significant associations of a history of trauma with the mode of birth, pregnancy and/or childbirth complications for the mother and the baby, and experiences of obstetric violence during labour, but not with preterm birth and medical procedures during labour. However, while some associations were significant in the presence of any type of past traumatic event, others were notable only in relation to specific types of abuse in the past. As for the cumulative effect of the lifetime traumatic types experienced, we found that the more types of abuse women had experienced, the higher were the risks for caesarean birth, pregnancy and/or childbirth complications for the mother and the baby, and encountering instances of obstetric violence, while we did not see these associations in relation to preterm birth and medical procedures during labour.

Although, following our hypothesis, we found that the more types of abuse women had experienced, the higher were the odds of them giving birth via caesarean, among the individual types of abuse this was true only for the survivors of physical abuse, but not those with sexual and/or child abuse in the past. This finding is not supported by previous evidence of an increased likelihood for a non-obstetrically indicated CB or an elective CB among adult sexual abuse survivors, but not those with a history of physical abuse at any age or child sexual abuse [28]. In a longitudinal study from Norway, on the other hand, women with a history of child abuse more frequently reported fear of childbirth and the wish for CB during second pregnancy [29]. Similar to our results, having a history of violence in a Swedish population increased the woman’s risk of having a CB [30]. These contradictory findings might shed light on the lack of consistency in the measurement of physical abuse experience, where timing, continuity, and the level of closeness with the aggressor might have different impacts on women and their childbirth experiences. As there is an overall lack of evidence on the effects of different types of past traumatic events on mode of birth, further studies are warranted.

Next, our results demonstrate that women with a history of any type of abuse are more likely to face complications for maternal health during pregnancy and/or childbirth. Furthermore, the more types of lifetime experiences of abuse women reported, the higher was the risk for more severe complications. These findings align with previous research of detrimental effects of trauma on maternal and child health during the perinatal period [18, 19]. Moreover, discoveries from neurobiology suggest that the abuse survivors are prone to hypervigilance due to more sensitive amygdala pathway [31], which may affect the perception of the severity of complications during pregnancy and/or childbirth among women with traumatic experiences. As we lack the objective information regarding the nature of the complications for maternal health, we are limited in the interpretation of the results in terms of the potential biological mechanisms underlying these associations. Nevertheless, these findings highlight the importance of trauma-informed maternity care and the need to discuss all the potentially disturbing events during pregnancy, birth, and postpartum period with women for them to feel safe, involved in the decision-making process, and confident that their concerns are heard, no matter whether they are driven by the objective or subjective criteria.

As for the complications for the baby’s health, these association were significant only for the child abuse survivors. Prolonged maternal child abuse is associated with neuroendocrine abnormalities, such as cortisol and oxytocin dysregulation [32], which can contribute to birth complications such as preterm delivery [33] and low birth weight [34]. Thus, these results underscore the potential intergenerational impact of childhood adversity and the need for comprehensive care and support for both mother and baby in these cases.

The complications for the child’s health do not seem to include preterm birth in our study, as we did not see any differences in the risks of preterm birth for women with any type or number of lifetime traumatic types experienced, contrary to our hypothesis. This finding contributes to the inconsistent evidence from the epidemiological review where among the six studies that met the inclusion criteria, in three women with a history of child sexual abuse had significantly higher odds of preterm birth, while the other three studies did not observe any statistical differences between women with and without a history of trauma [21]. It also seems to contradict our own finding of higher rates of pregnancy/childbirth complications among women with a history of child abuse. Prospective studies with more detailed reports of child abuse and objective records of preterm birth are needed to reveal more consistent results.

Similarly, our hypothesis regarding medical procedures was not supported, as we saw no increased risk of any medical procedures during labour in relation to any type of abuse or the number of lifetime traumatic types experienced in the past. This finding may suggest that, contrary to qualitative reports of abuse survivors’ preference for epidural analgesia [4], women with a history of physical, sexual and/or child abuse are not subjected to amniotomy, use of synthetic oxytocin, epidural analgesia, and episiotomy more often than women without such experiences.

Finally, there were several robust findings for the experiences of instances of obstetric violence during labour, where the risk of encountering at least one type of disrespect and abuse during labour was significantly higher among women with a history of physical and child, but not sexual abuse. This is an important discovery, which seems to contradict the existing body of literature highlighting the risks of re-traumatisation during childbirth for the survivors of sexual assault [4–7].However, due to the limitation of unknown timing of the types of the abuse in our study that we discuss further, the group with a history of child abuse may also include childhood sexual abuse survivors. Furthermore, this finding may indicate that women with a history of physical abuse and adverse childhood experiences other than sexual abuse have been overlooked in the research of perinatal outcomes. Thus, it is of paramount importance to take into account that any types of past traumatic experiences may elevate women’s sensitivity to their care providers’ behaviour during childbirth, which should be reflected in trauma-informed training of maternity care professionals.

Among the types of obstetric violence, the highest risk was for experiencing bullying and verbal abuse for both physical and child abuse survivors, while the risk for threats and accusations as well as denial of pain relief was higher only for women with a history of child abuse. This finding is in line with previous evidence of elevated lifetime risk of encountering violent behaviour for women with a history of abuse [26, 27]. Moreover, it could be a potential pathway moderating the significant association of past traumatic experiences with more severe symptoms of CB-PTSD we found in this cohort [9].

Several plausible mechanisms underlying the association between trauma history and obstetric violence can be considered. Firstly, it is possible that women with a history of trauma may have significant biological alterations of their stress-response systems that make them more sensitive to abusive behaviours during the childbirth process. Indeed, neurobiological research suggests that child abuse is strongly associated with dysregulation of the hypothalamic-pituitary-adrenal (HPA) axis and increased sensitivity of amygdala response which has long-term effects on stress response [31, 35]. Moreover, there is evidence of alterations in emotional and threatening words processing in individuals with previous traumatic experience [36], which is supported by the strongest associations with bullying and verbal abuse in our study.

Secondly, women with a history of trauma may exhibit heightened distrust or fear in healthcare settings that may impede their sense of self-efficacy, trigger dissociative states, and impair the process of communicating their needs to the healthcare providers [37]. As a result, women who are most prone to experience acute distress during obstetric procedures may be at risk of being subjected to more invasive procedures and interventions [38]. On the contrary, women who are more aware of their needs as the survivors of abuse, may be more vocal about them and refuse from some of the procedures, routinely performed by the midwives, thus, causing their disapproval and potential abusive behaviour in response [39].

Moreover, the organisational and systemic factors within healthcare settings may contribute to the occurrence of obstetric violence among women with a history of trauma due to inadequate training or lack of awareness among healthcare providers regarding trauma-informed care [7]. Considering that healthcare providers may not have access to a woman’s complete history, adopting a proactive approach and implementing universal precautions among clinicians and other perinatal professionals can create a safe and supportive environment that acknowledges and addresses the possibility of hidden trauma [4].

Notably, there were no statistical differences in the rates of the Kristeller manoeuvre use between the groups with and without a history of trauma, with an overall prevalence under 7%. However, the general trend is disturbing as during the pandemic this number was at 4.8% [40] and more than twice lower (3%) in the study among Russian women in 2020 [23]. Despite the WHO recommendations to avoid this practice which presents a serious risk for maternal and child health [41], it is still used by some healthcare providers. Further educational work among both policy makers, clinicians, and expecting parents is essential to eliminate this outdated practice that can physically and mentally traumatise all women, regardless of their previous status in relation to trauma.

### Strengths and limitations

This work is among the first studies that combines investigation of potential different effects of past physical, sexual and/or child abuse, their cumulative effect on multiple obstetric outcomes and their subtypes, namely multiple common medical procedures and different types of instances of obstetric violence occuring during labour. The strengths of our study encompass a considerable sample size, a well-designed study protocol, and the meticulous control of potential demographic and obstetric confounders. Nonetheless, it is imperative to recognize and carefully consider several important limitations when interpreting our results.

First, our research lacks objective data on the history of trauma and childbirth experiences, relying solely on self-reported information. Particularly, we have not asked to clarify the specific complications during pregnancy/childbirth the participants might have experienced, but only the level of their severity (none/minor/major), thus, we solely rely on their subjective perception of the events. This limitation is commonly encountered in perinatal studies, especially in countries where comprehensive registry-based data is not readily available. Moreover, both for our predictors (three types of abuse) and one of our outcomes (instances of obstetric violence) there are normally no objective records at all, especially in Russia where reports of abuse are strongly stigmatised, thus, making self-reports the only source of information.

Second, we did not inquire about the specific timing of the lifetime traumatic types experienced. As a result, instances of physical, sexual, and child abuse may have coincided with one another.

Third, contrary to global reports of 20–30% lifelong prevalence of physical, sexual, and child abuse among women [1–3], in our sample the estimates varied between 7 and 16.5% for all types of traumatic experiences. This may be due to high general stigmatisation of gendered sexual and intimate crimes [42, 43]. Particularly, in Russia violence against women is often narrated in the context of victim-blaming and cultural norms, preventing Russian women from disclosing domestic violence even to healthcare providers [44]. Thus, despite the anonymity in our survey, the reports of past traumatic events may not reflect the genuine picture and affect the reliability of the results.

Finally, the online data collection introduced sampling bias due to limited access to internet-connected devices, potentially excluding participants from certain regions of Russia. Recruitment via social media platforms may have resulted in a higher participation rate among women who are active online, potentially excluding marginalised and high-risk populations [45]. Moreover, the sample primarily comprised highly educated married women, thus, limiting the generalizability of the findings [46]. Further studies should include mothers from low-income groups to provide a more representative understanding of the childbirth experience in Russia.

## Conclusions

In conclusion, this study provides insights into the childbirth experiences of women with a history of physical, sexual, and/or child abuse. The findings demonstrate that women with any type of past abuse were at higher risk for maternal complications during pregnancy/childbirth, while the risks for encountering instances of obstetric violence were higher for physical and child abuse survivors, risks of cesarean birth were higher only for women with past physical abuse, and risks of complications during pregnancy/childbirth for the baby were elevated for women with child abuse experiences. There was also a cumulative effect of the types of lifetime trauma experienced for all the outcomes except for medical procedures during childbirth and preterm birth. These findings underscore the need for further research to better understand the underlying mechanisms linking different types of trauma history to perinatal outcomes. They further highlight the need to raise awareness that not only history of sexual abuse, but also other past adversities may affect women’s perception of the complications during childbirth as well as elevate their sensitivity to the care providers’ behaviour during labour, which should be reflected in trauma-informed training of maternity care professionals. Finally, while there were no significant associations of any type of past abuse with any type of medical procedures, we found strong effects of physical and child abuse on the risks of encountering instances of obstetric violence during labour. Thus, the present results confirm the evidence from the qualitative study of Montgomery and colleagues suggesting that it might not be the events of the childbirth that trigger memories of past abuse but rather the way the medical procedures are performed and communicated to the women [4]. Systematic thorough collection of women’s life history of trauma during antenatal visits and implementation of the principles of trauma-informed, respectful maternity care can work towards mitigating the risks of childbirth complications, obstetric violence, re-traumatisation, and CB-PTSD for women with a history of physical, sexual, and/or child abuse, fostering a more compassionate and sensitive approach to childbirth, and ultimately promoting positive birth experiences and maternal well-being.

## Data Availability

We are ready to provide anonymized dataset, syntaxes, and the survey form (in Russian) by a reasonable request. All requests should be directed to Dr. Anna Suarez via email anna.suarez.fig@gmail.com.

## Abbreviations

WHO: World Health Organization
PTSD: Posttraumatic Stress Disorder
CB: PTSD–Childbirth–Related Posttraumatic Stress Disorder
CB: Caesarean Birth
OR: Odds Ration
CI: Confidence Interval
HPA axis: Hypothalamic–Pituitary–Adrenal axis

## References

[1] Sardinha L, Maheu-Giroux M, Stöckl H, Meyer SR, García-Moreno C (2022). Global, regional, and national prevalence estimates of physical or sexual, or both, intimate partner violence against women in 2018. Lancet.

[2] Pan Y, Lin X, Liu J, Zhang S, Zeng X, Chen F (2021). Prevalence of childhood sexual abuse among women using the Childhood Trauma Questionnaire: a Worldwide Meta-Analysis. Trauma Violence Abuse.

[3] World Health Organization. Child maltreatment [Internet]. [cited 2023 May 7]. Available from: https://www.who.int/news-room/fact-sheets/detail/child-maltreatment.

[4] Montgomery E, Pope C, Rogers J (2015). The re-enactment of childhood sexual abuse in maternity care: a qualitative study. BMC Pregnancy Childbirth.

[5] Halvorsen L, Nerum H, Oian P, Sørlie T (2013). Giving birth with rape in one’s past: a qualitative study. Birth Berkeley Calif.

[6] Berman Z, Thiel F, Kaimal AJ, Dekel S (2021). Association of sexual assault history with traumatic childbirth and subsequent PTSD. Arch Womens Ment Health.

[7] van der Hulst LAM, Bonsel GJ, Eskes M, Birnie E, van Teijlingen E, Bleker OP (2006). Bad experience, good birthing: Dutch low-risk pregnant women with a history of sexual abuse. J Psychosom Obstet Gynaecol.

[8] Ayers S, Bond R, Bertullies S, Wijma K (2016). The aetiology of post-traumatic stress following childbirth: a meta-analysis and theoretical framework. Psychol Med.

[9] Suarez A, Yakupova V (2023). Past traumatic life events, Postpartum PTSD, and the role of Labor Support. Int J Environ Res Public Health.

[10] Seng JS, Low LK, Sperlich M, Ronis DL, Liberzon I (2009). Prevalence, trauma history, and risk for posttraumatic stress disorder among nulliparous women in maternity care. Obstet Gynecol.

[11] de Oliveira AGES, Reichenheim ME, Moraes CL, Howard LM, Lobato G (2017). Childhood sexual abuse, intimate partner violence during pregnancy, and posttraumatic stress symptoms following childbirth: a path analysis. Arch Womens Ment Health.

[12] Vogel TM, Homitsky S (2020). Antepartum and intrapartum risk factors and the impact of PTSD on mother and child. BJA Educ.

[13] Størksen HT, Garthus-Niegel S, Vangen S, Eberhard-Gran M (2013). The impact of previous birth experiences on maternal fear of childbirth. Acta Obstet Gynecol Scand.

[14] Garthus-Niegel S, Knoph C, von Soest T, Nielsen CS, Eberhard-Gran M (2014). The role of labor pain and overall birth experience in the development of posttraumatic stress symptoms: a longitudinal cohort study. Birth Berkeley Calif.

[15] Beck CT (2004). Birth trauma: in the eye of the beholder. Nurs Res.

[16] Perera E, Chou S, Cousins N, Mota N, Reynolds K (2023). Women’s experiences of trauma, the psychosocial impact and health service needs during the perinatal period. BMC Pregnancy Childbirth.

[17] Viirman F, Hesselman S, Poromaa IS, Svanberg AS, Wikman A (2023). Overall childbirth experience: what does it mean? A comparison between an overall childbirth experience rating and the Childbirth Experience Questionnaire 2. BMC Pregnancy Childbirth.

[18] Souch AJ, Jones IR, Shelton KHM, Waters CS (2022). Maternal childhood maltreatment and perinatal outcomes: a systematic review. J Affect Disord.

[19] Auger N, Low N, Lee GE, Ayoub A, Luu TM (2022). Pregnancy outcomes of women hospitalized for physical assault, sexual assault, and intimate Partner violence. J Interpers Violence.

[20] Henriksen L, Schei B, Vangen S, Lukasse M (2014). Sexual violence and mode of delivery: a population-based cohort study. BJOG Int J Obstet Gynaecol.

[21] Wosu AC, Gelaye B, Williams MA (2015). Maternal history of childhood sexual abuse and preterm birth: an epidemiologic review. BMC Pregnancy Childbirth.

[22] World Health Organization. The prevention and elimination of disrespect and abuse during facility-based childbirth: WHO statement [Internet]. World Health Organization. ; 2014 [cited 2023 May 16]. Report No.: WHO/RHR/14.23. Available from: https://apps.who.int/iris/handle/10665/134588.

[23] Yakupova V, Suarez A (2022). Postpartum PTSD and birth experience in russian-speaking women. Midwifery.

[24] Reed R, Sharman R, Inglis C (2017). Women’s descriptions of childbirth trauma relating to care provider actions and interactions. BMC Pregnancy Childbirth.

[25] Bohren MA, Vogel JP, Hunter EC, Lutsiv O, Makh SK, Souza JP (2015). The mistreatment of women during Childbirth in Health facilities globally: a mixed-methods systematic review. PLoS Med.

[26] Aakvaag HF, Thoresen S, Wentzel-Larsen T, Dyb G (2017). Adult Victimization in Female Survivors of Childhood Violence and abuse: the contribution of multiple types of violence. Violence Women.

[27] Perera D, Munas M, Swahnberg K, Wijewardene K, Infanti JJ, On Behalf Of The Advance Study Group null (2022). Obstetric violence is prevalent in Routine Maternity Care: a cross-sectional study of Obstetric Violence and its Associated factors among pregnant women in Sri Lanka’s Colombo District. Int J Environ Res Public Health.

[28] Schei B, Lukasse M, Ryding EL, Campbell J, Karro H, Kristjansdottir H (2014). A history of abuse and operative delivery – results from a European Multi-country Cohort Study. PLoS ONE.

[29] Lukasse M, Vangen S, Øian P, Schei B (2011). Fear of childbirth, women’s preference for cesarean section and childhood abuse: a longitudinal study. Acta Obstet Gynecol Scand.

[30] Finnbogadóttir H, Baird K, Thies-Lagergren L (2020). Birth outcomes in a Swedish population of women reporting a history of violence including domestic violence during pregnancy: a longitudinal cohort study. BMC Pregnancy Childbirth.

[31] Forster GL, Simons RM, Baugh LA. Revisiting the Role of the Amygdala in Posttraumatic Stress Disorder. The Amygdala - Where Emotions Shape Perception, Learning and Memories. InTech. [Internet]. [cited 2023 Dec 15].10.5772/67585.

[32] McGowan PO, Sasaki A, D’Alessio AC, Dymov S, Labonté B, Szyf M (2009). Epigenetic regulation of the glucocorticoid receptor in human brain associates with childhood abuse. Nat Neurosci.

[33] Buss C, Entringer S, Reyes JF, Chicz-DeMet A, Sandman CA, Waffarn F (2009). The maternal cortisol awakening response in human pregnancy is associated with the length of gestation. Am J Obstet Gynecol.

[34] Zhang G, Srivastava A, Bacelis J, Juodakis J, Jacobsson B, Muglia LJ. Genetic studies of gestational duration and preterm birth. Best Pract Res Clin Obstet Gynaecol. 2018;52:33–47. 10.1016/j.bpobgyn.2018.05.003 PMC6290110 30007778

[35] Teicher MH, Samson JA, Anderson CM, Ohashi K (2016). The effects of childhood maltreatment on brain structure, function and connectivity. Nat Rev Neurosci.

[36] Iffland B, Eilers R, Rosner R, Renneberg B, Steil R, Neuner F (2023). Differentiated processing of emotional cues in adolescents and young adults with ICD-11 PTSD and complex PTSD after child abuse. Brain Behav.

[37] Leeners B, Görres G, Block E, Hengartner MP (2016). Birth experiences in adult women with a history of childhood sexual abuse. J Psychosom Res.

[38] Jonsdottir IV, Sigurdardottir S, Halldorsdottir S, Jonsdottir SS (2022). We experienced lack of understanding in the healthcare system. Experiences of childhood sexual abuse survivors of the childbearing process, health and motherhood. Scand J Caring Sci.

[39] Shabot SC (2021). Why ‘normal’ feels so bad: violence and vaginal examinations during labour – a (feminist) phenomenology. Fem Theory.

[40] Yakupova V, Suarez A, Kharchenko A (2021). Birth experience, Postpartum PTSD and Depression before and during the pandemic of COVID-19 in Russia. Int J Environ Res Public Health.

[41] World Helath Organization. WHO recommendations: intrapartum care for a positive childbirth experience [Internet]. [cited 2023 Mar 13]. Available from: https://www.who.int/publications-detail-redirect/9789241550215. 30070803

[42] Kennedy AC, Prock KA (2018). I still feel like I am not normal: a review of the role of Stigma and Stigmatization among female survivors of child sexual abuse, sexual assault, and intimate Partner violence. Trauma Violence Abuse.

[43] Gurieva SD, Kazantseva TV, Mararitsa LV, Gundelakh OE (2022). Social Perceptions of Gender Differences and the subjective significance of the gender inequality issue. Psychol Russ.

[44] Lokhmatkina NV, Kuznetsova OY, Feder GS (2010). Prevalence and associations of partner abuse in women attending Russian general practice. Fam Pract.

[45] Yakupova VA, Suarez AD, Shraibman LA (2023). Socioeconomic risk factors for Postpartum Depression and Postpartum Post-traumatic stress disorder. Russ Psychol J.

[46] Odintsova MA, Lubovsky DV, Ivanova PA, Gusarova ES (2022). Special characteristics of the resilience of Russian families in the Face of Modern challenges (a preliminary study). Psychol Russ.

